# Insertion Torque, Removal Torque, and Resonance Frequency Analysis Values of Ultrashort, Short, and Standard Dental Implants: An In Vitro Study on Polyurethane Foam Sheets

**DOI:** 10.3390/jfb14010010

**Published:** 2022-12-23

**Authors:** Luca Comuzzi, Margherita Tumedei, Tea Romasco, Morena Petrini, Kelvin I. Afrashtehfar, Francesco Inchingolo, Adriano Piattelli, Natalia Di Pietro

**Affiliations:** 1 Independent Researcher, San Vendemiano-Conegliano Veneto, 31020 Treviso, Italy; 2 Department of Medical, Surgical, and Dental Sciences, University of Milan, 20122 Milan, Italy; 3 Department of Medical, Oral and Biotechnological Sciences, “G. d’Annunzio” University of Chieti-Pescara, 66013 Chieti, Italy; 4 Center for Advanced Studies and Technology-CAST, “G. d’Annunzio” University of Chieti-Pescara, 66013 Chieti, Italy; 5 Clinical Sciences Department, College of Dentistry, Ajman University, Ajman Emirate P.O. Box 346, United Arab Emirates; 6 Department of Reconstructive Dentistry and Gerodontology, School of Dental Medicine, University of Bern, 3010 Berne, Switzerland; 7 Department of Interdisciplinary Medicine, University of Bari “Aldo Moro”, 70124 Bari, Italy; 8 School of Dentistry, Saint Camillus International University of Health and Medical Sciences, 00131 Rome, Italy

**Keywords:** short implants, ultrashort implants, implant stability, insertion torque, removal torque, atrophic jaws

## Abstract

Short implants were introduced to reduce morbidity, treatment duration, and complex bone regeneration interventions in atrophic jaws and to improve patient-reported outcomes. This study aimed to determine the insertion torque (IT), removal torque (RT), and resonance frequency analysis (RFA) values of ultrashort (3 mm length), short (7 mm length), and standard implants (10 mm length) inserted in 1-, 2-, 3-, and 4-mm thickness polyurethane sheets with densities of 10, 20, and 30 pounds per cubic foot (PCF). Standard-length implants were the gold standard (control). Overall, short-length implant IT values were higher or similar to the control in most experimental conditions. Those inserted into a 3 mm/30 PCF lamina showed the highest IT values, whereas 5 mm diameter ultrashort-length implants inserted into 2 and 3 mm/20 PCF laminas were higher than other implants. RT values followed the same trend and RFA values were more appreciable in short- and standard-length implants in all the scenarios. However, ultrashort-length implants reached a primary stability comparable to that of standard implants in lower thicknesses. In conclusion, although further studies are needed to corroborate this in vitro model with preclinical and clinical studies, our data shed light on short- and ultrashort-length implants geometries to a potential application in critical atrophy of the posterior jaws.

## 1. Introduction

The definition of short- and ultrashort- (or extra-short) length implants is still debated in the literature. Most of the authors agree in defining as “short” those implants with a length ranging from 5 to 8 mm [1,2,3,4,5,6,7,8,9,10,11,12]. Recently, Lombardo et al. (2020) defined “ultrashort“as implants with a length less than or equal to 5 mm [13], reporting a survival rate of 96.6% for single-crown restorations supported by short- and ultrashort-length implants in a 3-year follow-up study. Regarding this type of implant, Pistilli et al. (2020) reported no implant/prosthetic failure with 4 mm length implants in a 7-year follow-up case [14], while Felice et al. reported that short-length implants after 5 years from being loaded had significantly lower marginal bone loss (MBL) in respect to standard-length implants [15]. In 2019, an in vitro study using polyurethane foam models with different densities and thicknesses tested 2.5 mm and 3.5 mm length implants [16]. These studies have shown that success rates comparable to those of long implants can be achieved with short implants by decreasing the lateral forces to the prosthesis, eliminating cantilevers, increasing implant surface area, and improving the implant to abutment connection.

Bone atrophy of the alveolar ridges follows after tooth loss, especially in the jaw posterior regions. Hence, appropriate quantity and quality of the alveolar bone are needed to ensure the correct tridimensional implant positioning and obtain optimal esthetic and functional outcomes [14,17,18]. Several possible alternatives to treat bone atrophy are available, such as autogenous bone block onlays or inlays, guided bone regeneration (GBR) procedures, inferior alveolar nerve repositioning, distraction osteogenesis, sinus floor elevation (SFE), ridge-splitting, and bone expansion [19,20,21,22]. However, all these techniques could present drawbacks, such as extreme technical demands, high morbidity incidence, complications (up to 20% of the cases), or failures. Moreover, these drawbacks imply unpredictable outcomes, high costs, and prolonged treatment time depending on the surgical procedure. Hence, there is need for a less invasive treatment option in areas of poor bone quantity and quality. As an alternative, short- and ultrashort-length implants have been introduced to reduce rehabilitative times and costs, avoid the possible use of grafting procedures [23] and other more invasive surgical treatments, and reduce patient discomfort and morbidity [5,19,20]. Due to the biological and economic advantages in using this type of fixture to prosthetically rehabilitate an atrophic jaw, several studies have reported the survival rates of fixed prostheses implants supported at various follow-up points, showing their efficacies [13,14,15].

Thus, the aim of the present in vitro study was to evaluate the in vitro biomechanical behavior of ultrashort- (3 mm length), short- (7 mm length), and standard-length (10 mm length) implants in different simulated clinical scenarios. This objective was performed by comparing the insertion torque (IT), removal torque (RT), and resonance frequency analysis (RFA) values of these implants inserted in polyurethane foam models of different thicknesses and densities to obtain additional information about shorter implants for corroborating their possible clinical application in the critical atrophy of posterior jaws, instead of using a more complicated vertical ridge augmentation procedure.

## 2. Materials and Methods

### 2.1. Implants, Polyurethane Foam Sheets and Study Design

**Figure 1:**
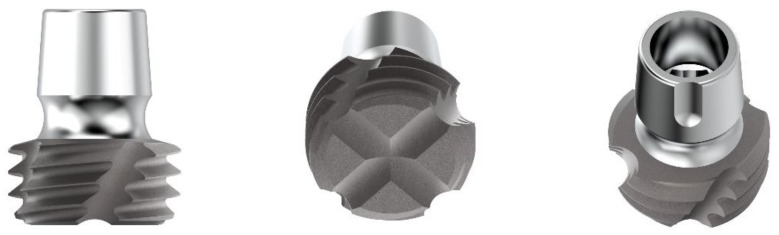
Details of ultrashort-length implants tested in the present investigation. From the left: lateral, bottom (apex), and top (connection) views.

**Figure 2:**
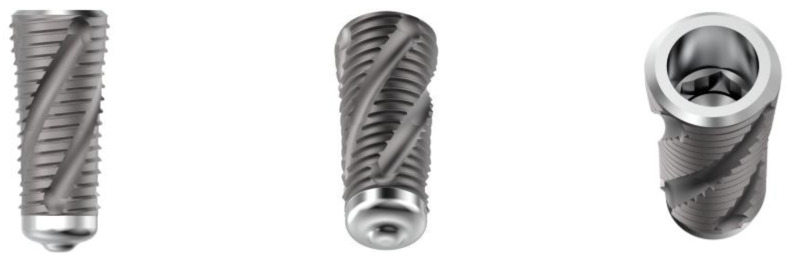
Details of short-length implants for maxillary sinus lift tested in the present investigation. From the left: lateral, bottom (apex), and top (connection) views.

**Figure 3:**
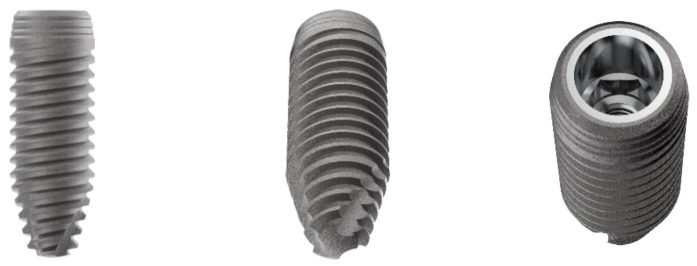
Details of standard-length implants tested in the present investigation. From the left: lateral, bottom (apex), and top (connection) views.

The characteristics of the different implants used for this in vitro investigation are listed as follows: ultrashort-length implants (ACY40030N200C Cyroth Ø 4 × 3 mm OsteoPore CC, and ACY50030N200C Cyroth Ø 5 × 3 mm, OsteoPore CC, AoN Implants S.r.l., Grisignano di Zocco, Vicenza, Italy) with a diameter of 4 and 5 mm and a length of 3 mm (Figure 1), short-length implants for maxillary sinus lift (ALC42070N200C SLC Ø 4.2 × 7 mm, OsteoPore CC, AoN Implants S.r.l., Grisignano di Zocco, Vicenza, Italy) with a diameter of 4.2 mm and a length of 7 mm (Figure 2), and standard-length implants (ACY40100N200C Cyroth Ø 4 × 10 mm, OsteoPore CC, AoN Implants S.r.l., Grisignano di Zocco, Vicenza, Italy) with a diameter of 4 mm and a length of 10 mm (Figure 3). Ultrashort-length implants have a cylindrical macromorphology and a flat apex with grooves to make the liquids flow. Standard-length implants have a cylindrical macromorphology and a conical apex, while short-length implants have a cylindrical morphology apically and conically at the coronal level (tapered morphology). All the previous implants have a conical self-locking Cone Morse connection (RevCon, AoN Implants S.r.l., Grisignano di Zocco, Vicenza, Italy). Concerning the microtopography, all these types of implants have been subjected to the OsteoPore treatment, obtained by double acidification of the part of the thread, in order to create surface structures and roughness at the micro level. This treatment was followed by washing and final decontamination by plasma. This process provides all these implants with the same surface roughness, with micro-pits separated by distances in the order of 2 μm (μm), making them extremely efficient for activating platelet aggregation and clot retention at the implant site [24,25,26,27,28,29,30].

**Figure 4:**
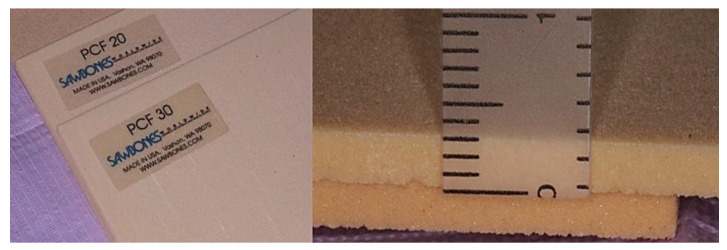
Details of the different polyurethane laminas of 20 and 30 PCF density used in the in vitro simulation. Right image, a 3 mm thick lamina.

**Figure 5:**
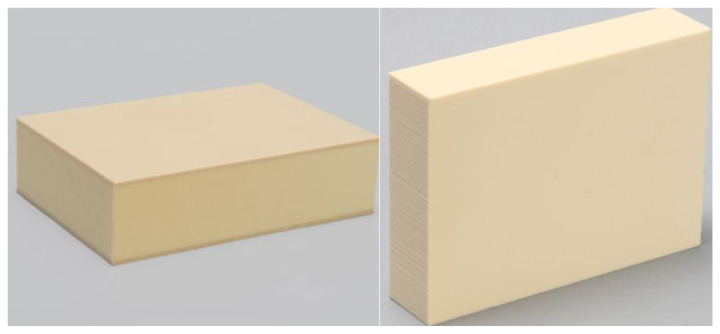
Details of 4 mm thick blocks with (on the **left**) or without (on the **right**) 1 mm thick cortical sheet with a density of 30 PCF used in the in vitro simulation.

The American Society for Testing and Materials (ASTM F-1839-08) (“Standard specification for Rigid Polyurethane Foam for Use as a Standard Material for Test Orthopedic Devices for Instruments”) has recognized polyurethane foam sheets as alternative materials for biomechanical tests, even for dental implant evaluations. This material does not replicate human bone structure, yet it displays consistent mechanical characteristics similar to bone tissue. Additionally, it results in being very reliable and easy to use, requiring no special handling, and it is characterized by linearly elastic and constitutive isotropic symmetry [31,32]. As previously reported also by Comuzzi et al. [16], polyurethane foam sheets represent the most suitable material for in vitro use, simulating the consistency and different densities of bone tissue to compare dental implants and bone screws. In particular, less than 3 mm thick sheets simulate recurrent critical clinical conditions, such as ridge atrophy and maxillary sinus pneumatization. Artificial bone has the convenience of presenting pronounced mechanical characteristics, avoiding human variables or particular handling and preservation treatments whilst preserving similar properties to natural bone. Nowadays, it is also preferred to cadaver or animal bones for ethical reasons. In this study, 1, 2 and 3 mm thick laminas with densities of 20 and 30 pounds per cubic foot (PCF) (corresponding to a density of 0.32 g/cm^3^, similar to the D2 bone type and 0.48 g/cm^3^, similar to D1 bone type, respectively) (Figure 4), and also 4 mm thickness blocks with densities of 10 and 20 PCF (corresponding to a density of 0.16 g/cm^3^, similar to the D3 bone type, and D2 bone density, respectively), with or without 1 mm thick cortical sheet with a density of 30 PCF (Figure 5), were used to test the implants. In particular, the polyurethane foam sheets presented the following sizes: 13 cm × 18 cm × 4 mm (concerning the bone blocks); 13 cm × 18 cm × 1 mm (concerning the cortical bone sheets on the blocks); 13 cm × 18 cm × 1 mm (concerning the laminas of 20 and 30 PCF in density), 13 cm × 18 cm × 2 mm (concerning the lamina of 20 PCF in density), and 13 cm × 18 cm × 3 mm (concerning the laminas of 20 and 30 PCF in density). All the polyurethane foam sheets were purchased from Sawbones Europe AB (Malmö, Sweden).

**Figure 6:**
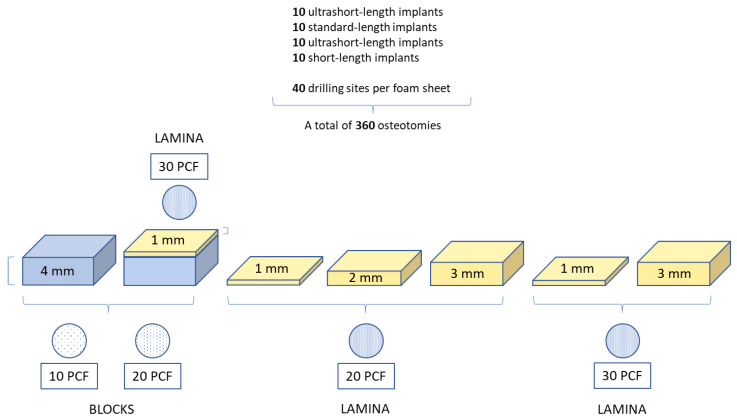
Summary of the osteotomies performed and the study design.

A total of 360 osteotomies (10 for each implant type) were performed on the different polyurethane foam models. In this way, 40 drilling sites were obtained for each sheet (Figure 6).

### 2.2. Drilling Protocol

The investigation was conducted by a single operator (LC). Implants were positioned in the polyurethane blocks and laminas of any thickness and density, following the corresponding manufacturer’s protocol.

Short-length implants were previously inserted using a lanceolate drill, and then a 2.2 mm and a 3.2 mm drill with the use of a surgical implant motor (Chiropro, Bien Air, Bienne, Switzerland) at 100 rpm.

The ultrashort-length implant protocol was performed using a lanceolate drill before a 2 mm drill. Finally, 4 mm diameter ultrashort-length implants were positioned with a 3.2 mm drill, and 5 mm diameter ultrashort-length implants with a 4.1 mm drill, using the same surgical implant motor at 100 rpm.

Regarding standard-length implants, the manufacturer’s protocol was performed using a lanceolate drill, then a 2 mm drill, and finally a 3.2 mm drill, using the surgical implant motor at 100 rpm.

**Figure 7:**
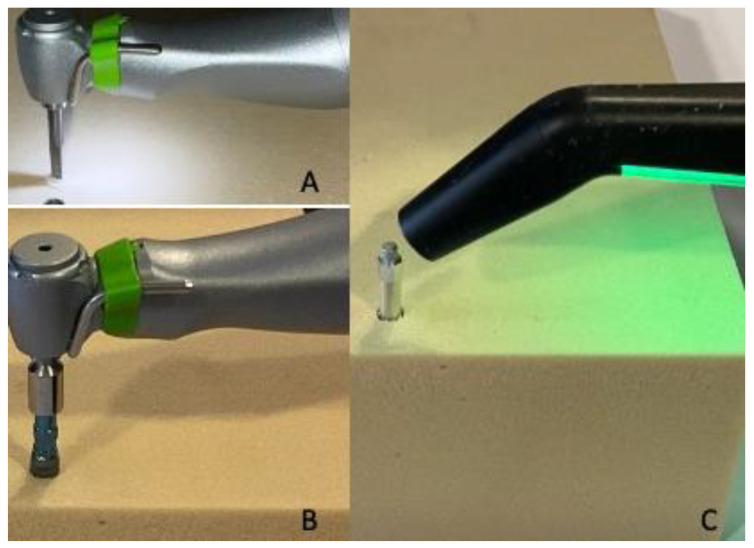
(**A**,**B**) Details of site preparation and implant insertion. (**C**) RFA measurement of dental implant stability after screw positioning.

The investigation was conducted to determine the insertion torque and removal torque strength values of the four tested implants inserted into polyurethane foam models of different thicknesses and densities. In particular, after implant positioning at 20–30 rpm, the final 1 mm IT and RT values were recorded by dynamometric analysis using a calibrated torque meter during screw positioning. As already described in our previous study [16], the RFA values were measured by a dedicated device Smartpeg n.78, Ostell Inc., Göteborg, Schweden, recording the implant stability quotient (ISQ) in two different orientations at 90 degrees (Figure 7).

### 2.3. Statistical Analysis

Power analysis and sample size planning were performed using the ANCOVA statistical test (effect size: 0.264, α err: 0.05; power (1-β): 0.95; numerator df: 10; number of groups: 7; number of covariates: 9), using the program G*Power 3.1.9.7. The minimum total sample size necessary to achieve a statistically significant output was 360 implant sites.

A one-way analysis of variance (ANOVA) followed by Tukey’s post hoc test was performed to evaluate the statistical significance of the study variables. The study data were analyzed using the statistical software package GraphPad 9.0 (Prism, San Diego, CA, USA). The statistical significance was set at *p* < 0.05.

## 3. Results

The experimental results related to the implants’ IT, RT, and RFA values evaluation and comparison are reported in Table 1.

These values were obtained from independent measurements acquired by the different implants inserted in each artificial bone condition.

**Figure 8:**
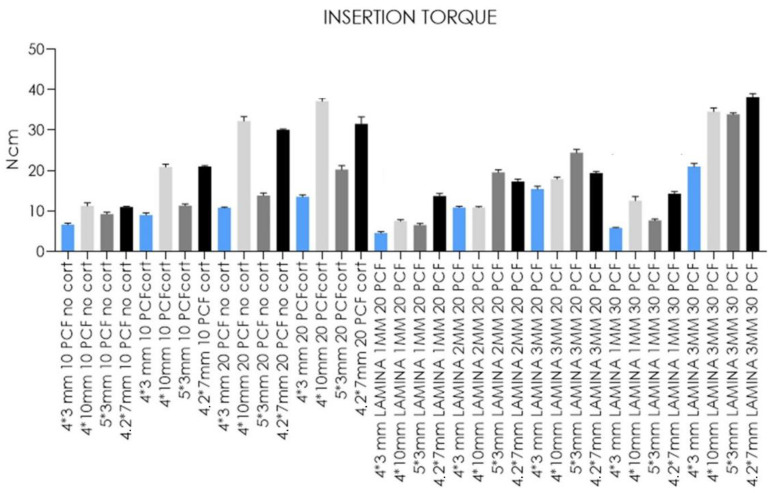
Bar graphs related to the distribution of insertion torque values expressed by all the implant types in the different artificial bone conditions. Data are expressed as means ± SD. Data not statistically significant are indicated as ns (not significant), while all other values are significant with *p* < 0.05.

**Figure 9:**
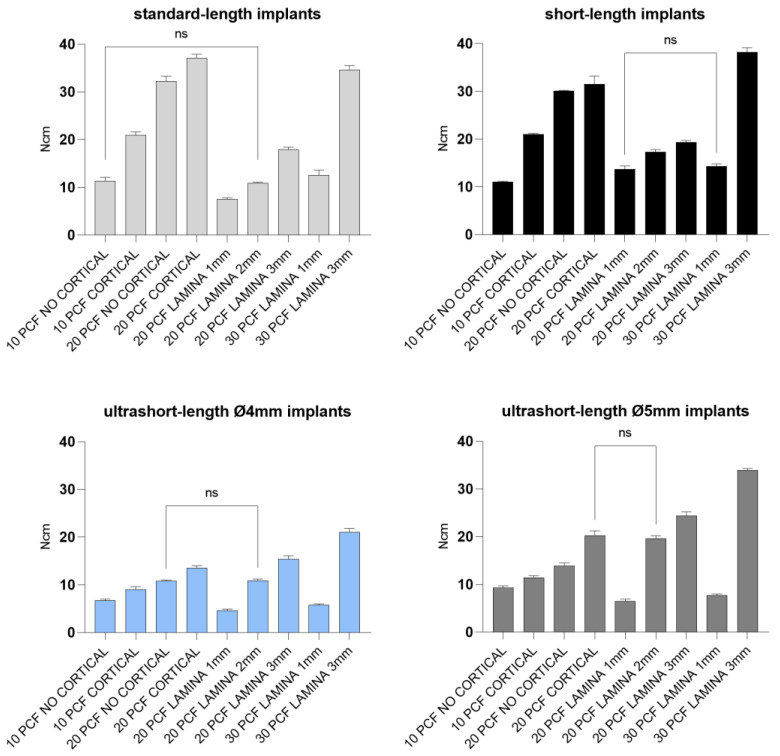
Bar graphs related to the distribution of insertion torque values expressed by each implant type in the different artificial bone conditions. Data were expressed as means ± SD. Data not statistically significant are indicated as ns (not significant), while all other values are significant with *p* < 0.05.

In Figure 8 and Figure 9 report the comparison of the IT values expressed by all the implant types and the IT values expressed by single types of implants, respectively. 

Concerning the data reported from standard-length implants, significantly higher IT values were found in the block of 20 PCF density with the cortical sheet (37.1 Ncm). In comparison, the 1 mm thick lamina of 20 PCF density showed the lowest values (7.5 Ncm). No statistical differences were detected between measurements taken after insertion into the block of 10 PCF density without the cortical sheet and the 2 mm lamina of 20 PCF density. Short-length implant IT values, instead, ranged from 38.2 and 11 Ncm when inserted in the 3 mm thick lamina of 30 PCF density and the block of 10 PCF density without the cortical sheet, respectively. The values reported for the 1 mm thick lamina of 20 PCF density, and the 1 mm thick lamina of 30 PCF showed no statistical differences. Both of the ultrashort-length implants tested reported significantly higher IT values when inserted in the 3 mm thick lamina of 30 PCF density (21 and 33.9 Ncm for 4- and 5-mm diameter ultrashort-length implants, respectively) and the lowest values in the 1 mm thick lamina of 20 PCF density (4.6 and 6.5 Ncm for 4- and 5-mm diameter ultrashort-length implants, respectively). Despite this, IT values of 4 mm diameter implants for the block of 20 PCF density without the cortical sheet and the 2 mm lamina of 20 PCF density resulted similar results. In comparison, 5 mm diameter implant IT values for the block of 20 PCF with the cortical sheet and the 2 mm thick lamina of 20 PCF density also had comparable results.

**Figure 10:**
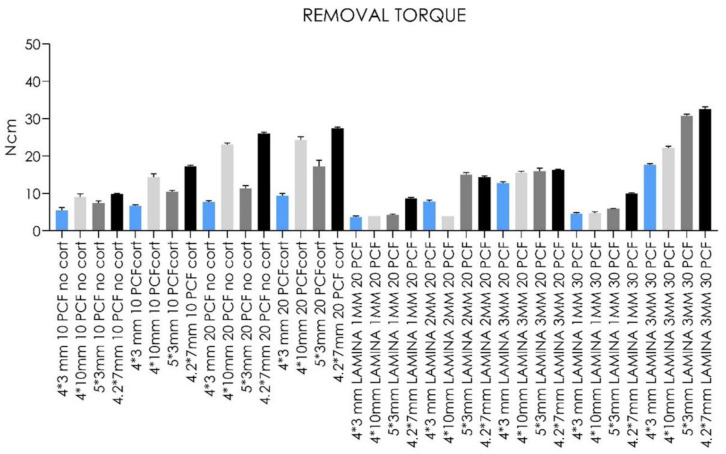
Bar graphs related to the distribution of removal torque values expressed by all the implant types in different artificial bone conditions. Data are expressed as means ± SD. Data not statistically significant are indicated as ns (not significant), while all other values are significant with *p* < 0.05.

**Figure 11:**
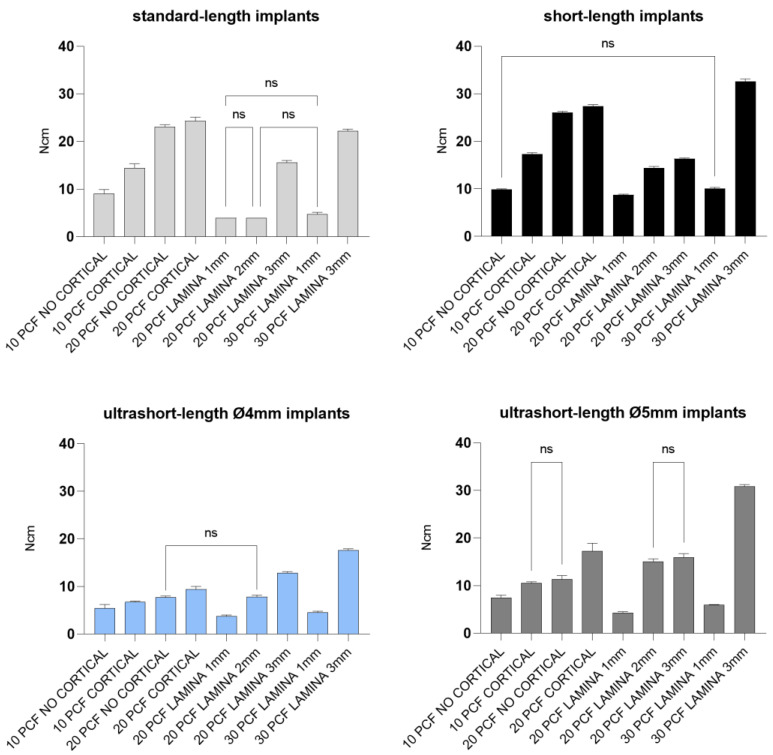
Bar graphs related to the distribution of removal torque values expressed by each implant type in the different artificial bone conditions. Data are expressed as means ± SD. Data not statistically significant are indicated as ns (not significant), while all other values are significant with *p* < 0.05.

Removal torque values were about 1–12 Ncm lower than the corresponding IT values for each tested implant (Table 1, Figure 10 and Figure 11). 

In line with this, less force was needed to remove implants of low-density and thickness compared with high-density or thickness sheets, irrespective of implant design.

In general, the higher the density of the sheet, the higher the IT and RT values for all types of implants with cortical presence.

**Figure 12:**
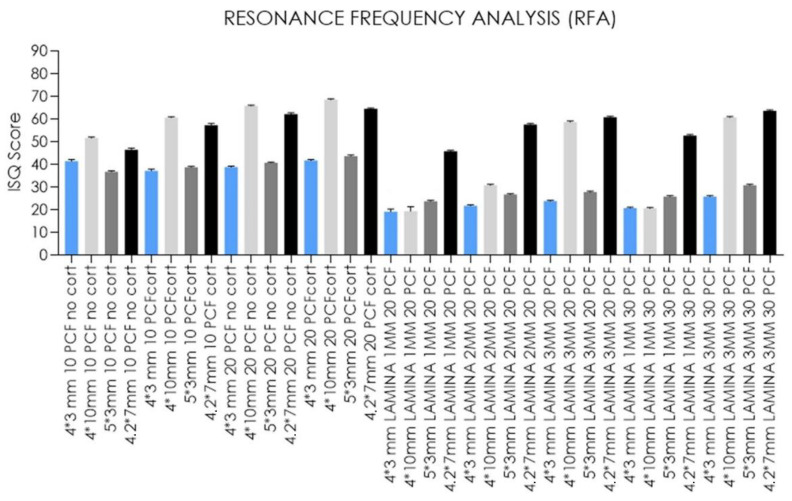
Bar graphs related to the distribution of resonance frequency analysis values expressed by all the implant types in the different artificial bone conditions. Data are expressed as means ± SD. Data not statistically significant are indicated as ns (not significant), while all others are significant with *p* < 0.05.

**Figure 13:**
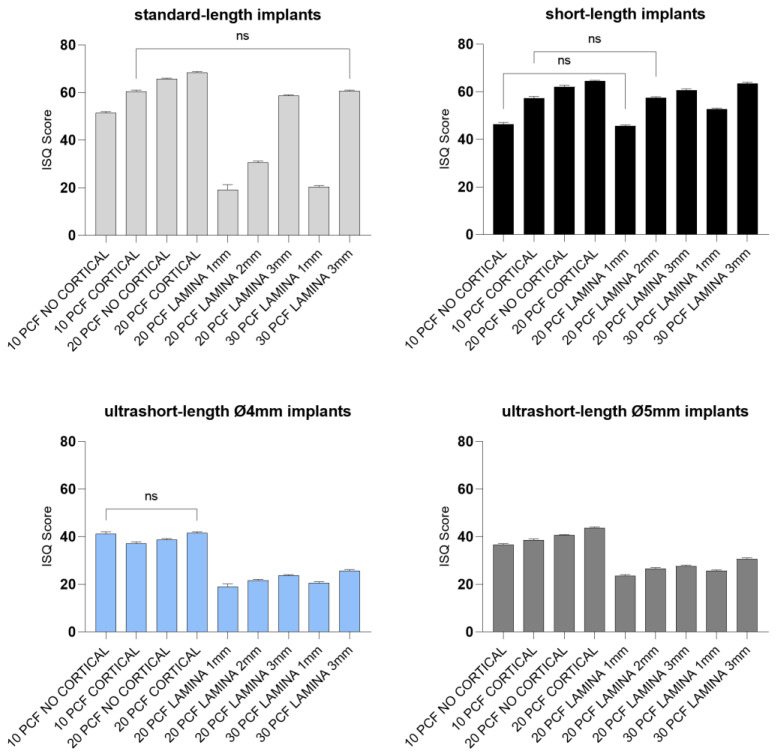
Bar graphs related to the distribution of resonance frequency analysis values expressed by each implant type in the different artificial bone conditions. Data are expressed as means ± SD. Data not statistically significant are indicated as ns (not significant), while all others are significant with *p* < 0.05.

Regarding RFA values, standard-length implants showed ISQ values ranging from 19.2 to 68.4, with the highest results for 4 mm thickness blocks, especially for the block of 20 PCF density with the cortical sheet, and the lowest for the 1 mm thick lamina of 20 PCF density. As described for standard-length implants, short-length implants also reported comparable primary stability results, ranging from 45.6 to 63.5 ISQ, when inserted in thinner polyurethane foam sheets. Ultrashort-length implants instead showed lower ISQ values (19–41.6 and 23.6–43.6 for 4- and 5-mm diameter ultrashort-length implants, respectively) in almost all densities and thicknesses in respect to other implants, reporting the lowest values in the 1 mm thick lamina of 20 PCF in density and the highest in the block of 20 PCF density with the cortical sheet (Figure 12 and Figure 13). 

However, much lower values (ISQ 19.2, 20.4, and 30.7), similar or even lower to those registered for both ultrashort-length implants, were found for standard-length implants inserted in 1- and 2-mm thick laminas of 20 and 30 PCF density (Table 1).

## 4. Discussion

Implant performances and results are related to the implant geometry, surface characteristics, loading conditions, bone quantity and quality, biomechanical anchorage of the implant threads to the peri-implant mineralized bone, surgical techniques, and the right fitting into the host bone [33,34,35,36].

Furthermore, in vitro studies help to comprehend the biomechanical forces involved in the placement of implants and could suggest potential occurrences in a clinical context [37]. In particular, artificial bone could help avoid the variability within and among species.

In the case of an edentulous and severely atrophied posterior jaw, the presence of sufficient quantity and good quality of bone is mandatory for correct implant insertion and an optimal aesthetic result [17]. For this purpose, several treatment techniques are already well-established, although they report a high rate of morbidity and complication.

In the last few years, short and ultrashort-length implants have been proposed as effective alternatives to more complicated reconstructive bone surgery procedures. In fact, using implants of such lengths implies a less invasive approach and reduces cost, healing time, peri-operative morbidity, and patient discomfort [5,13,22,23,38].

Moreover, no significant differences between 4 mm length implants and longer implants have been reported in terms of implant survival rates in the literature. On the other hand, short-length implants presented significantly lower marginal bone resorption rates and fewer biological and prosthetic complications [5,14,15,23,37,39,40].

In de Oliveira et al.’s in vitro study [41], IT values and primary stability similar to standard-length conventional implants were presented for short-length implants without comparing different bone densities. The literature reported that tapered and larger implants had shown better primary stability in terms of ISQ values and also higher ISQ values than parallel-walled implants. In addition, short-length implants showed higher ISQ values than even ultrashort-length implants [42] and increased primary stability, especially in low-quality bone [43].

Moreover, our in vitro results (Table 1 and Figure 8, Figure 9, Figure 10, Figure 11, Figure 12 and Figure 13) found higher IT values for short-length implants in almost all experimental conditions, except for the 10 PCF block without the cortical sheet and the 20 PCF blocks with or without cortical. Indeed, the results were comparable to standard-length implants, whereas the corresponding RT values were higher in all blocks and laminas.

Conversely, the lowest values were registered for ultrashort-length implants inserted in the 1 mm thick lamina of 20 PCF density. However, in the latter case and in the 1 mm thick lamina of 30 PCF density, the primary stability appeared to be higher than or comparable to standard-length implant ISQ values. The IT values for ultrashort-length implants showed the best results at higher foam densities. In particular, the IT value for 5 mm diameter ultrashort-length implants inserted in 2- and 3-mm thick laminas of 20 PCF density reached 24.4 and 19.6 Ncm, thus higher than other implant values. For this reason, it could be stated that ultrashort-length implants showed better primary stability than other implants, even with low ISQ values.

Although short-length implant geometry reported a primary stability of 45.6–63.5 ISQ, particularly in case of lower artificial bone height, ultrashort-length implants resulted in comparable or higher primary stability obtained compared to standard-length implants in 1 mm thick polyurethane foam laminas of 20 and 30 PCF density, laying the foundations for possible use in cases of critical posterior mandible height, instead of more invasive augmentation procedures.

However, in clinical situations, many biological factors affect the primary stability and physiological and molecular events of the bone’s healing to produce phenomena such as bone resorption, neoformation, and remodeling, leading to secondary stability. Regarding the limitations of the present study, we can report that only the mechanical aspects of the effect of surface treatment were evaluated against the biological factors, such as bone response, individual characteristics, local variations in human bone and the surgical technique, which also influence primary stability in a clinical situation. Regarding the material (synthetic bone blocks) used, inhomogeneity due to the presence of fat, bone marrow, and blood inside real human bone is challenging to simulate in a foam model. However, to the end of this work, it was assumed that the contributions of these components are negligible.

The favorable results showed by short-length implants were probably correlated to their tapered shape, while ultrashort-length implants are cylinder-shaped, although they presented the same surface treatment. For this reason, higher friction between the implant and the polyurethane foam material was produced. The similar values obtained by 4- and 5-mm diameter ultrashort-length implants probably support the hypothesis that a moderate positive correlation between ISQ values and length and a weak correlation with diameter, which has already been reported [42]. Maximum bone stress resulted in being independent of implant length, in contrast with implant width, which is fundamental to optimizing loading stress distribution. Most of the stress appeared to be distributed to the bone adjacent to the initial implant threads [33]. Overall, from the aforementioned results, it can be stated that IT, RT, and ISQ values increased, even with the use of ultrashort-length implants, as bone density and thickness of the polyurethane foam sheets increased and in relation to the presence of a cortical sheet over them, confirming previously published studies, in which density and primary stability were directly proportional [35,36,43,44,45,46].

Our analysis on 10 PCF polyurethane sheets reproduced a critical clinical condition corresponding to in vivo D3 bone density, and 20 PCF sheets corresponded to in vivo D2 bone density. At the same time, the 30 PCF setting was more similar to the most common D1 bone jaw density according to the Misch classification [47]. Thus, the strength of this study was the possibility of resembling the relative results of real-world conditions.

On the contrary, another limitation of this study could be that the study design provided only an analysis of the influence of the implant length and microtopography on the insertion torque, removal torque, and primary stability, whereas the discrepancies in implant macromorphology could constitute an additional factor that might occur in different performance of the tested implants.

Despite this, the authors could speculate that when standard-length implants can be used, the use of short-length or ultrashort-length implants could be neglected. However, the possibility that short-length implants could be chosen to achieve better primary stability by bicortical fixation can be glimpsed. Moreover, when less bone is available in the mandibular posterior alveolar ridge, even in the lowest bone density cases, ultrashort-length implants could provide sufficient primary stability instead of performing vertical augmentation surgery, which requires higher costs, could affect the osseointegration process with a longer healing period, and implies higher peri-operative morbidity and patient discomfort. However, the ultrashort-length implants’ primary stability in vitro must still be improved by developing the macro-design and micro-surface. In the present case, a conical shape seemed more suitable than a cylindrical one. Additionally, a different pitch of the threads may allow for a better grip of the fixture.

Lastly, the literature about current in vivo studies seems to support prosthetic compensation of biomechanical behavior and masticatory forces in the posterior jaw, producing a prosthesis joint in the case of short-/ultrashort-length implants to reduce mechanically adverse events [45,48].

## 5. Conclusions

Within the limits of the present in vitro study, the insertion and removal torque values increased as the artificial bone density increased in all implant types tested; however, consistently lower removal torque values were obtained whenever the implants were extracted. Therefore, the benefits reported for short implants in the literature, with the corroboration of this in vitro study, such as reduction of the entire treatment and surgical intervention duration, cost-effectiveness, and the avoidance of complex regenerative procedures, could be extrapolated to other simple and minimally invasive approaches, such as the studied 3 mm long ultrashort implants. Nevertheless, ex vivo and in silico studies with adequate sample sizes on this matter are required before preclinical and clinical trials can be considered.

## Figures and Tables

**Table 1:** Statistic values of IT, RT, and RFA related to the different experimental conditions tested for each type of implant (**A**: 4 mm diameter ultrashort-length implants; **B**: standard-length implants; **C**: 5 mm diameter ultrashort-length implants; **D**: short-length implants).

**IT**	**10 PCF**								**20 PCF**								**20 PCF**				**20 PCF**				**20 PCF**				**30 PCF**				**30 PCF**			
	**BLOCK NO CORT**				**BLOCK CORT**				**BLOCK NO CORT**				**BLOCK CORT**				**LAMINA 1 MM**				**LAMINA 2 MM**				**LAMINA 3 MM**				**LAMINA 1 MM**				**LAMINA 3 MM**			
	**A**	**B**	**C**	**D**	**A**	**B**	**C**	**D**	**A**	**B**	**C**	**D**	**A**	**B**	**C**	**D**	**A**	**B**	**C**	**D**	**A**	**B**	**C**	**D**	**A**	**B**	**C**	**D**	**A**	**B**	**C**	**D**	**A**	**B**	**C**	**D**
Mean	6.7	11.3	9.3	11.0	9.0	20.9	11.4	21.0	10.8	32.2	13.9	30.1	13.5	37.1	20.2	31.5	4.6	7.5	6.5	13.7	10.9	10.9	19.6	17.3	15.4	17.9	24.4	19.3	5.8	12.6	7.7	14.3	21.0	34.6	33.9	38.2
SD	0.3	0.8	0.4	0.2	0.6	0.7	0.4	0.2	0.2	1.1	0.6	0.1	0.5	0.8	1.0	1.7	0.3	0.3	0.4	0.7	0.3	0.2	0.6	0.5	0.7	0.5	0.8	0.4	0.2	1.0	0.3	0.5	0.8	0.9	0.4	0.9
SErr	0.1	0.3	0.1	0.1	0.2	0.2	0.1	0.1	0.1	0.4	0.2	0.0	0.2	0.2	0.3	0.5	0.1	0.1	0.1	0.2	0.1	0.1	0.2	0.2	0.2	0.2	0.3	0.1	0.0	0.3	0.1	0.2	0.2	0.3	0.1	0.3
Lower 95% CI	6.4	10.7	9.0	10.9	8.6	20.4	11.1	20.9	10.7	31.4	13.4	30.0	13.2	36.6	19.5	30.3	4.3	7.3	6.3	13.2	10.7	10.8	19.1	16.9	14.9	17.5	23.8	19.0	5.7	11.8	7.5	13.9	20.4	33.9	33.6	37.5
Upper 95% CI	6.9	11.8	9.6	11.2	9.4	21.4	11.7	21.2	11.0	33.0	14.3	30.2	13.9	37.7	21.0	32.8	4.8	7.7	6.8	14.2	11.1	11.0	20.0	17.7	15.9	18.3	25.0	19.6	6.0	13.3	7.9	14.7	21.5	35.2	34.2	38.8
**RT**	**10 PCF**								**20 PCF**								**20 PCF**				**20 PCF**				**20 PCF**				**30 PCF**				**30 PCF**			
	**BLOCK NO CORT**				**BLOCK CORT**				**BLOCK NO CORT**				**BLOCK CORT**				**LAMINA 1 MM**				**LAMINA 2 MM**				**LAMINA 3 MM**				**LAMINA 1 MM**				**LAMINA 3 MM**			
	**A**	**B**	**C**	**D**	**A**	**B**	**C**	**D**	**A**	**B**	**C**	**D**	**A**	**B**	**C**	**D**	**A**	**B**	**C**	**D**	**A**	**B**	**C**	**D**	**A**	**B**	**C**	**D**	**A**	**B**	**C**	**D**	**A**	**B**	**C**	**D**
Mean	5.4	9.0	7.4	9.9	6.7	14.4	10.5	17.3	7.7	23.1	11.3	26.0	9.4	24.3	17.2	27.4	3.7	3.9	4.3	8.7	7.8	3.9	15.0	14.4	12.8	15.6	15.9	16.3	4.5	4.7	5.9	10.0	17.6	22.2	30.8	32.6
SD	0.8	0.9	0.6	0.1	0.2	0.9	0.3	0.3	0.3	0.4	0.8	0.3	0.6	0.8	1.7	0.3	0.3	0.0	0.2	0.2	0.4	0.0	0.6	0.3	0.3	0.4	0.8	0.2	0.3	0.4	0.1	0.3	0.3	0.4	0.4	0.5
SErr	0.3	0.3	0.2	0.0	0.1	0.3	0.1	0.1	0.1	0.1	0.3	0.1	0.2	0.3	0.5	0.1	0.1	0.0	0.1	0.1	0.1	0.0	0.2	0.1	0.1	0.1	0.3	0.1	0.1	0.1	0.0	0.1	0.1	0.1	0.1	0.2
Lower 95% CI	4.8	8.4	7.0	9.8	6.6	13.8	10.3	17.0	7.5	22.8	10.7	25.8	9.0	23.7	16.0	27.2	3.5	3.9	4.1	8.5	7.6	3.9	14.6	14.1	12.6	15.3	15.4	16.1	4.3	4.4	5.8	9.8	17.4	22.0	30.5	32.2
Upper 95% CI	6.0	9.7	7.8	10.0	6.9	15.0	10.7	17.5	8.0	23.4	11.9	26.3	9.9	24.9	18.4	27.7	3.9	3.9	4.4	8.9	8.1	3.9	15.4	14.6	13.0	15.8	16.5	16.4	4.8	5.0	6.0	10.1	17.9	22.5	31.1	33.0
**RFA**	**10 PCF**								**20 PCF**								**20 PCF**				**20 PCF**				**20 PCF**				**30 PCF**				**30 PCF**			
	**BLOCK NO CORT**				**BLOCK CORT**				**BLOCK NO CORT**				**BLOCK CORT**				**LAMINA 1 MM**				**LAMINA 2 MM**				**LAMINA 3 MM**				**LAMINA 1 MM**				**LAMINA 3 MM**			
	**A**	**B**	**C**	**D**	**A**	**B**	**C**	**D**	**A**	**B**	**C**	**D**	**A**	**B**	**C**	**D**	**A**	**B**	**C**	**D**	**A**	**B**	**C**	**D**	**A**	**B**	**C**	**D**	**A**	**B**	**C**	**D**	**A**	**B**	**C**	**D**
Mean	41.3	51.5	36.6	46.3	37.1	60.5	38.6	57.2	38.7	65.7	40.6	62.0	41.6	68.4	43.6	64.4	19.0	19.2	23.6	45.6	21.6	30.7	26.6	57.5	23.7	58.6	27.6	60.7	20.6	20.4	25.7	52.6	25.7	60.6	30.6	63.5
SD	0.8	0.5	0.5	0.8	0.7	0.5	0.5	0.8	0.5	0.4	0.3	0.7	0.5	0.5	0.5	0.4	1.2	2.1	0.5	0.5	0.5	0.5	0.4	0.4	0.4	0.4	0.5	0.5	0.4	0.5	0.4	0.5	0.4	0.4	0.5	0.5
SErr	0.2	0.1	0.1	0.2	0.2	0.1	0.1	0.2	0.2	0.1	0.1	0.2	0.1	0.2	0.2	0.1	0.4	0.7	0.1	0.1	0.1	0.2	0.1	0.1	0.1	0.1	0.1	0.2	0.1	0.2	0.1	0.2	0.1	0.1	0.1	0.2
Lower 95% CI	40.7	51.2	36.3	45.7	36.6	60.2	38.3	56.6	38.3	65.4	40.4	61.5	41.3	68.0	43.2	64.1	18.1	17.7	23.3	45.3	21.3	30.3	26.2	57.2	23.4	58.2	27.3	60.3	20.2	20.0	25.4	52.2	25.4	60.2	30.3	63.1
Upper 95% CI	41.8	51.8	36.9	46.9	37.6	60.8	38.9	57.8	39.0	65.9	40.8	62.4	41.9	68.7	43.9	64.6	19.9	20.7	23.9	45.9	21.9	31.0	26.9	57.8	23.9	58.9	27.9	61.0	20.9	20.8	26.0	52.9	25.9	60.9	30.9	63.8

## Data Availability

Data is contained within the article and available on request from the corresponding author.
